# Transactions College of Physicians

**Published:** 1849-04

**Authors:** 


### TRANSACTIONS OF COLLEGE OF PHYSICIANS.

The Summary of the Transactions of the College of Physicians of Philadelphia, from September 6th, 1848, to January 2d, 1849, has been received, and we have looked through its pages with great satisfaction. Besides what we have transferred to our own pages, it embraces a report on Practical Medicine, a memoir of the late Dr. Henry Neill, a report on Midwifery, and a variety of miscellaneous matter, of decided interest. We always expect the appearance of this Summary with pleasure, and never fail to derive instruction from its discussions and reports. It is a summary of facts especially interesting to the physician in practice, the members of the College themselves belonging to that class.

